# Contribution of Neuroepigenetics to Huntington’s Disease

**DOI:** 10.3389/fnhum.2017.00017

**Published:** 2017-01-30

**Authors:** Laetitia Francelle, Caroline Lotz, Tiago Outeiro, Emmanuel Brouillet, Karine Merienne

**Affiliations:** ^1^Department of NeuroDegeneration and Restorative Research, University Medical Center GoettingenGoettingen, Germany; ^2^CNRS UMR 7364, Laboratory of Cognitive and Adaptive Neurosciences, University of StrasbourgStrasbourg, France; ^3^Commissariat à l’Energie Atomique et aux Energies Alternatives, Département de Recherche Fondamentale, Institut d’Imagerie Biomédicale, Molecular Imaging Center, Neurodegenerative diseases Laboratory, UMR 9199, CNRS Université Paris-Sud, Université Paris-SaclayFontenay-aux-Roses, France

**Keywords:** neuroepigenetics, Huntington’s disease, epigenomics, transcriptomics, neuronal activity, HDAC inhibitors, neurodegenerative diseases

## Abstract

Unbalanced epigenetic regulation is thought to contribute to the progression of several neurodegenerative diseases, including Huntington’s disease (HD), a genetic disorder considered as a paradigm of epigenetic dysregulation. In this review, we attempt to address open questions regarding the role of epigenetic changes in HD, in the light of recent advances in neuroepigenetics. We particularly discuss studies using genome-wide scale approaches that provide insights into the relationship between epigenetic regulations, gene expression and neuronal activity in normal and diseased neurons, including HD neurons. We propose that cell-type specific techniques and 3D-based methods will advance knowledge of epigenome in the context of brain region vulnerability in neurodegenerative diseases. A better understanding of the mechanisms underlying epigenetic changes and of their consequences in neurodegenerative diseases is required to design therapeutic strategies more effective than current strategies based on histone deacetylase (HDAC) inhibitors. Researches in HD may play a driving role in this process.

## Introduction

Huntington’s disease (HD) is a genetic disease affecting preferentially medium spiny neurons (MSN) of the striatum. Increasing numbers of studies provide evidence for altered epigenetic regulations in HD. Here, after a summary of general epigenetic mechanisms in neurons, we review the state-of-the-art of epigenetic changes in HD. We discuss the mechanisms underlying these changes and their consequences, particularly on expression of neuronal identity genes. Current challenges to improve our understanding of the role of epigenetic mechanisms in HD are discussed, including the development of new technologies to profile neuronal epigenomes at single-cell or cell-type specific levels. Epigenetic modifications represent promising therapeutics. We discuss the need of identifying new epigenetic targets for therapy, representing alternatives to histone deacetylase (HDAC) inhibitors.

### Epigenetic Mechanisms: General Rules

**FIGURE 1:**
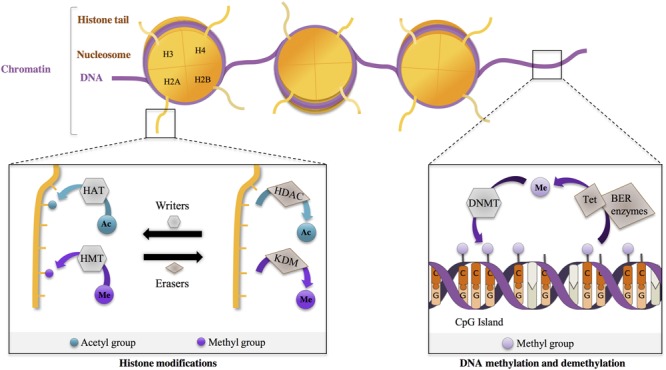
**Epigenetic regulations are dynamic.** Chromosomal DNA is wrapped around histone octamers (comprising dimers of H2A, H2B, H3, H4), forming nucleosomes, the core unit of chromatin. Protruding N-terminus histone tails can undergo post-translational modifications (PTM) that influence chromatin state (e.g., relaxed vs. compact state) Epigenetic writers add PTM on histone tail residues. For example, histone acetyltransferases (HAT) add acetyl- (Ac) groups, and histone methyltransferases (HMT) add methyl- (Me) groups. In a dynamic manner, these marks are removed by epigenetic erasers, such as histone deacetylases (HDAC) and lysine demethylases (KDM), removing, respectively, Ac- and Me-groups. DNA methylation at cytosines, particularly at CpG dinucleotides, is performed by DNA methyltransferases (DNMT). DNA demethylation involves hydroxylation and oxydation of methylated cytosines by Tet proteins (Tet), which are then repaired by DNA repair mechanisms, including base excision repair (BER).

The current definition of epigenetics relates to “the study of phenomena and mechanisms that cause chromosome-bound, heritable changes to gene expression that are not dependent on changes to DNA sequence” (Deans and Maggert, 2015). Epigenetic mechanisms actually regulate several DNA/RNA-mediated processes, including transcription, DNA repair and DNA replication, through modulation of the structure of chromatin, a macromolecular complex of DNA, RNA and proteins such as histones. Two major epigenetic mechanisms influence chromatin structure: histone modifications and DNA methylation (Allis and Jenuwein, 2016) (**Figure 1**).

In the nucleus, DNA is wrapped around core particles of chromatin, the nucleosomes, which are formed of octamers of histones, including H2A, H2B, H3, and H4. Histones are subject to various post-translational modifications (PTM) such as acetylation, methylation, phosphorylation and ubiquitylation. These histone modifications modulate the degree of compaction of nucleosomes, thereby affecting chromatin accessibility to various factors, particularly transcriptional regulators. (Jenuwein and Allis, 2001; Borrelli et al., 2008; Wang and Jin, 2010; Tsompana and Buck, 2014; Xu et al., 2014). Importantly, histone residues can be modified in a combinatorial, reversible, and targeted manner (**Figure 1**). For instance, histone acetyltransferases (HAT) and HDAC are involved in acetylation and deacetylation of specific histone residues, respectively (**Figure 1**). Similarly, methylases and demethylases regulate the addition and removal of methyl groups on histone residues (**Figure 1**). The combinatorial, reversible and targeted nature of histone modifications is the basis of the so-called ‘histone code’. It permits to achieve specificity in the outcome, through the action of proteins interpreting the code (e.g., “readers”) (Jenuwein and Allis, 2001; Bannister and Kouzarides, 2011; Jones et al., 2016). One major outcome is transcription. General rules of the “transcriptional” histone code are relatively well defined. For instance, histone acetylation, whatever the histone or the residue modified, promotes relaxed chromatin and is associated with transcriptional activation, whereas histone methylation, depending on the specific residue that is modified, can lead to transcriptional repression or activation. For instance, H3K9 methylation is associated with transcription repression, whereas H3K4 methylation correlates with transcription activation. Moreover, the genome comprises different regulatory regions, including promoters and enhancers (i.e., regulatory regions distal to promoters) playing specific roles in transcriptional regulation. These regions display specific histone modifications. For instance, promoters and enhancers of active genes are enriched in trimethylated H3K4 (H3K4me3) and in acetylated H3K27 (H3K27ac) respectively, further illustrating the targeting precision permitted by the histone code.

DNA methylation, another important epigenetic mechanism, consists in adding a methyl group to cytosines by DNA methyltransferases (DNMT), particularly at C5 position of cytosine in cytosine-guanine dinucleotide sequences (CpG), creating 5-methylcytosine (5-mC). DNMT1 is implicated in the maintenance of methylation patterns during DNA replication. In contrast, DNMT3a and DNMT3b have been associated with *de novo* DNA methylation (Jeltsch, 2006; Feng et al., 2010). Though DNA methylation was long considered as a stable process, it is now clear that post-mitotic cells can undergo active DNA demethylation, through a mechanism implicating TET proteins (Pastor et al., 2013). TET proteins induce hydroxylation of 5-mC (leading to 5-hmc) and are further involved in their oxidation. Oxidized 5-hmC are then processed by DNA repair mechanisms and converted back to their unmethylated state (Guo et al., 2011; Feng et al., 2015). 5-hmC is particularly extended in neurons, suggesting that regulation of DNA methylation is highly dynamic in these cells (Song et al., 2011; Szulwach et al., 2011). 5-mC are bound by several classes of methyl-binding proteins (such as MeCP2), which associate with other protein partners, including HDAC, forming co-repressor complexes (Urdinguio et al., 2009; Du et al., 2015). DNA methylation at gene promoters, generally enriched in CpG sites and forming so-called CpG islands, is an important mechanism involved in gene repression (Deaton and Bird, 2011) (**Figure 1**).

The collection of epigenetic modifications (e.g., the epigenome) can be assessed at genome-wide scale, using next generation sequencing-based techniques such as chromatin-immunoprecipitation-sequencing (ChIP-seq). Generation and integration of epigenomic data with transcriptomic and/or functional data to fully decode epigenomes at cell-type specific level is a major current challenge that requires the development of new techniques and analysis methods (Marconett et al., 2013; Shin et al., 2014). In particular, methods to process low cell numbers/single-cell need to be developed as well as powerful bioinformatics tools (Bock et al., 2016).

#### Neuroepigenetics: Why are Neurons Specific?

Epigenetic mechanisms, interfacing individual genomes with environmental factors, are essential to the regulation of fundamental biological processes. Historically, epigenetic mechanisms have been primarily explored in the context of development, including cellular differentiation and the establishment of stable cellular identity (Holliday, 2006; Roth and Sweatt, 2011; Boland et al., 2014). In fact, cell state transitions during development require massive epigenetic changes, whose stabilization enables the maintenance of cell-type specific identity. The mechanisms involve the establishment of defined transcriptional programs. As a result, the epigenome is a gatekeeper of cell-type specific identity. However, this view of stable epigenetics has been challenged, due to evidence showing that massive and dynamic epigenetic changes can be implicated in the regulation of cellular activity. Such “plasticity” of epigenetic regulations is particularly critical to neuronal cell activity; hence the concept of neuroepigenetics (Borrelli et al., 2008; Riccio, 2010; Sweatt, 2013; Deans and Maggert, 2015).

Neuronal excitability is one major property of neuronal cells. In response to environmental experience, including learning, drug exposure, psychological and physical stress environmental signals, neurons undergo reversible transitions from resting to active (or excited) states, which influence synaptic plasticity and promote adaptive behavior, such as learning and memory (Sultan and Day, 2011; Sweatt, 2016). These processes are highly dynamic, and can also be long-lasting. Increasing evidence indicates that epigenetic mechanisms regulate the transition from resting to active neuronal state (Korzus, 2010; Landgrave-Gomez et al., 2015; Meadows et al., 2016).

Learning and memory processes are associated with synaptic plasticity, leading to the rapid formation of new synapses, which can be strengthened or lost over time. Synaptic plasticity correlates with dynamic changes at the level of histone modifications. Specifically, changes in acetylation of histones, including H3 and H4 have been associated with early formation of new synapses (Federman et al., 2009; Chatterjee et al., 2013; Graff and Tsai, 2013; Peixoto and Abel, 2013; Benito et al., 2015). Moreover, neuronal activity can be associated with memory storage and consolidation, which may involve long-term remodeling of neuronal networks at system levels. Epigenetic regulations have also been implicated in these processes. Upon learning paradigms, the chromatin in brain tissues implicated in memory formation and/or consolidation, such as the hippocampus and the cortex, undergoes extensive modifications, including increased histone acetylation and DNA methylation changes (Day and Sweatt, 2011; Bousiges et al., 2013; Zovkic et al., 2013; Halder et al., 2016). DNA methylation and histone modifications both control memory processes, through transcriptional effects that comprise the activation of synaptic plasticity genes, including immediate early genes (IEGs) like *Fos, Egr1*, or *Arc* (Minatohara et al., 2015; Thakurela et al., 2015). Remarkably, these transcriptional effects that are experience-driven and epigenetically regulated can be long lasting. Activation of specific signaling pathways, such as the cAMP/CREB/CREB-binding protein (CBP) pathway, is involved in the coupling between epigenetic and transcriptional responses, promoting the recruitment of protein complexes at target genes, which induces chromatin remodeling and drive transcription (Cedar and Bergman, 2009; Tie et al., 2014; Alberini and Kandel, 2015; Ortega-Martinez, 2015).

However, although tight coupling between neuronal-activity-regulated epigenetic and transcriptional changes remains a favored hypothesis, recent data, integrating epigenomic, and transcriptomic data suggest that the link between both events may not be as clear as anticipated (Lopez-Atalaya and Barco, 2014; Liu et al., 2015; Valor, 2015b). Recently, contextual fear conditioning in mice was used as a learning paradigm to examine the spatiotemporal correlation between epigenetic and transcriptional modifications in neurons (Halder et al., 2016). Specifically, DNA methylation and seven histone modifications were assessed at a genome-wide scale using hippocampal and cortical neurons of mice that were subjected to contextual fear conditioning. To specify the timing of epigenetic changes in these tissues, analyses were performed at different time points with respect to contextual fear conditioning, associated with different memory processes, including memory formation and consolidation. Transcriptomic analyses were also performed on the same tissues. Generally, histone modifications were extensively modulated during memory formation, and these widespread changes weakly correlated with gene-selective transcriptional changes. In contrast, DNA methylation changes were rather locus-specific and correlated with transcriptional changes, including splicing events (Lev Maor et al., 2015; Halder et al., 2016). Thus, the coupling between epigenetic and transcriptional changes may depend on chromatin modifications. Additional studies are required to specify causal relationship between the two events. Other neuronal cells/tissues and experimental paradigms may be used, which would permit to investigate the interplay between epigenetics and transcription in brain cells/tissues associated with additional brain functions, including other cognitive functions, motor functions and functions linked to emotion and motivation regulation.

Thus, it appears that epigenetic mechanisms in neurons not only control their identity, like in other cell types, but also regulate neuronal activation, including the ability to undergo dynamic plasticity in response to environmental signals. Then, the question arising is what is going on in pathological situations, when neuronal function is impaired? Does it result from altered activation capacity of affected neurons or from a loss of neuronal identity? Are impaired epigenetic regulations implicated in pathological processes? These questions are particularly relevant to neurodegenerative diseases where specific neuronal populations (or identities) are preferentially affected. In the following sections, we have chosen to focus on one such disease, HD, playing a pioneering role in the understanding of the epigenetic regulation mechanisms in brain diseases.

## Epigenetics in HD

Huntington’s disease is a neurodegenerative disease caused by an unstable expanded CAG repeats (>35–39 repeats) in the Huntingtin gene (*HTT*), which results in the production of mutant protein (mHtt) with a toxic polyglutamine (polyQ) tract (Landles and Bates, 2004). HD is characterized by specific symptoms, including motor impairment (e.g., chorea, bradykinesia, gait abnormalities, dystonia), cognitive deficits (motor skill learning deficits, planning, and attention troubles) and psychiatric alterations (depression, mania, apathy, suicide), usually appearing at adulthood (see as review, Roos, 2010). Since polyQ expansion toxicity is correlated with repeat size, HD patients with longer CAG expansions are more severely affected, showing earlier onset of symptoms and faster pathology progression. HD is characterized by a preferential and primary degeneration of two structures of the basal ganglia: the caudate nucleus and the putamen that form the neostriatum. However, additional brain regions, particularly the cortex, degenerate as the pathology progresses (Rosas et al., 2008). Remarkably, in HD striatum, selective neuronal populations, the GABAergic MSN, are more particularly vulnerable, whereas the large cholinergic interneurons, the medium size GABAergic interneurons and glial cells appear spared (Ferrante et al., 1987a,b; Cicchetti et al., 1996). From a biochemical point of view, polyQ-Htt presents a high propensity to misfold and aggregate, which leads to the formation of nuclear inclusions, particularly in neurons (DiFiglia et al., 1997; Li L. et al., 2016). These aggregates, a disease hallmark, recruit a number of proteins, including transcriptional regulators (Steffan et al., 2000; Arrasate and Finkbeiner, 2012).

A long period of neuronal dysfunction precedes the death of neurons sensitive to the HD mutation. Several cellular processes are believed to contribute to neuronal dysfunction, including Htt cleavage and aggregation, abnormal protein-protein interactions, dysfunctional calcium signaling, abnormal axonal transport, impaired energy metabolism, dysregulation of neuronal activity, and altered transcription (see as reviews Landles and Bates, 2004; Zuccato et al., 2010; Labbadia and Morimoto, 2013; Saudou and Humbert, 2016).

### The “Neuronal” Signature of the HD Transcriptome

Transcriptional dysregulation plays a central role in HD pathogenesis (Seredenina and Luthi-Carter, 2012). Major transcriptional changes have been reported in the brains of HD patients (Hodges et al., 2006). This is also observed in HD mouse models, including transgenic and knock-in mice (Luthi-Carter et al., 2000; Luthi-Carter et al., 2002; Zucker et al., 2005; Roze et al., 2008). Transcriptomic studies using mice showed that transcriptional changes are progressive, CAG repeat-length-dependent and most extended in the striatum (Desplats et al., 2006; Kuhn et al., 2007; Becanovic et al., 2010; Langfelder et al., 2016). In the striatum of HD models, transcriptional changes occur in both directions: many genes are down- and up-regulated (Seredenina and Luthi-Carter, 2012; Francelle et al., 2014). Remarkably, down-regulated genes display a neuronal signature, since decreased genes in HD striatum are enriched in genes that define striatal neuron identity and function, such as *Darpp32, Rgs9, Drd1* or *Drd2* (Hodges et al., 2006; Kuhn et al., 2007; Vashishtha et al., 2013; Achour et al., 2015; Langfelder et al., 2016). In the other affected brain regions such as the cortex, fewer genes are down-regulated. However, they also present a neuronal signature, reflecting tissue identity (Vashishtha et al., 2013; Langfelder et al., 2016).

Interestingly, a recent study using RNAseq revealed a developmental signature associated with differentially expressed genes in post-mortem prefrontal cortex of HD patients. Specifically, Hox genes and additional homeobox genes were re-expressed in HD brains, suggesting that the transcriptome of HD neurons resemble that of immature neurons (Labadorf et al., 2015). Thus, these results support the view that the transcriptional program involved in the maintenance of neuronal identity is impaired in HD neurons. Up-regulated genes in the striatum or cortex of HD patients were also enriched in biological processes linked to inflammation (Hodges et al., 2006; Labadorf et al., 2015). However, up-regulation of immune response genes is not that clear in mouse models (Achour et al., 2015; Langfelder et al., 2016). Thus, down- and up-regulated genes in HD brain tissues display distinct functional signatures, suggesting that different mechanisms may operate.

The mechanisms underlying mutant Htt-induced transcriptional effects are unclear. However, they are thought to involve both altered regulation of transcription regulators and histone-modifying enzymes.

### HDACi in HD: What are the Targets?

The hypothesis of an epigenetic origin of transcriptional dysregulation in HD, particularly implicating histone acetylation, has received increasing support over the years (Jaenisch and Bird, 2003; Lee et al., 2013; Glajch and Sadri-Vakili, 2015). The assumption that altered histone acetylation might contribute to HD was made in the early 2000s, when it was found that the HAT CREB-binding protein CBP is recruited into aggregates of mutant Htt, and that HDAC inhibitors (HDACi) improve phenotypes of drosophila and mouse models of HD (Nucifora et al., 2001; Steffan et al., 2001; Kazantsev et al., 2002). As a result, epigenetic strategies using HDACi to increase histone acetylation have been early considered for HD. Several preclinical studies have been performed using broad-spectrum HDACi (e.g., suberoxylanilide hydroxamic acid (SAHA), Trichostatin A (TSA), phenylbutyrate, sodium butyrate (NaB)] that target non-selectively HDAC of class I and II (Ferrante et al., 2003; Hockly et al., 2003; Gardian et al., 2005; Sadri-Vakili et al., 2007; Sharma and Taliyan, 2015b).

Histone deacetylase inhibitors improve some phenotypes of HD mice, including neuropathology and motor function. However, it is unclear whether beneficial effects require increased histone acetylation. Instead, studies suggest that the mechanism may involve acetylation of non-histone proteins. In support to this view, inactivation of a target of SAHA, *Hdac 4*, ameliorates neurodegeneration in HD mice through an apparently, transcription-independent mechanism, acting on mutant Htt aggregation process (Mielcarek et al., 2011). Non-histone-mediated beneficial effects of HDACi have also been documented in models of Parkinson disease (PD; Godena et al., 2014), suggesting common mechanisms between several neurodegenerative diseases.

New compounds have been developed in an attempt to generate more selective HDACi and with less toxic side effects (Herman et al., 2006; Thomas et al., 2008). The compound HDACi 4b, which was reported to ameliorate disease phenotype of HD mice, show high potency for inhibiting HDAC1 and HDAC3 (Thomas et al., 2008; Jia et al., 2012). However, physicochemical properties and metabolic profile of this compound were found suboptimal for investigation of HDAC inhibition in mice per oral administration (Beconi et al., 2012). The effect of RGFP966, an HDAC3-selective inhibitor, was recently investigated using HD mice (Jia et al., 2016). The results suggest that the compound limits glial cell response, diminishing markers of glial cell activation. Surprisingly however, a heterozygous inactivation of the *Hdac3* gene in HD mice did not ameliorate disease-related phenotypes (Moumne et al., 2012), suggesting that more than 50% knock-down of the *Hdac3* gene might be needed to see a beneficial effect. More recently, beneficial transgenerational effects have been reported using HDACi 4b in HD mice (Jia et al., 2015). Thus, despite some beneficial effects, the mode of action of HDACi in HD models remains elusive and may not systematically implicate histone- and transcription-dependent mechanisms.

It is still unclear whether expression of neuronal identity genes in HD brain tissues is restored upon HDACi treatment (Coppede, 2014; Wang et al., 2014; Sharma et al., 2015; Valor, 2015a). Clinical studies using HDACi are ongoing and the results are awaited. So far, Phase II studies provide indication for safety and tolerability of several compounds, including phenylbutyrate (Hogarth et al., 2007; Westerberg et al., 2015).

But what exactly is the status of the HD epigenome? Is acetylation the only histone modification impaired in HD affected tissues, and to which extent? Are acetylated histone residues all affected the same way by the HD mutation? Is DNA methylation also impaired? Is the chromatin structure globally altered and repressed or is it altered at specific genomic regions or gene loci? What is the underlying mechanism? What are the consequences of chromatin structures changes? Do they underlie transcriptional changes? Addressing these questions is certainly a prerequisite to the development of new epigenetic therapies for HD.

### HD Epigenome

Many studies have already been performed that attempt to address these issues (Lee et al., 2013; Valor and Guiretti, 2014; Glajch and Sadri-Vakili, 2015). It is expected that the use of genome-scale approaches, which has remained so far rather limited in the HD field, will improve our knowledge of HD epigenome as well as provide insights into the mechanism responsible for altered epigenetic regulation in HD.

#### Relationship between Epigenetic and Transcriptional Changes in HD

##### Histone acetylation

**FIGURE 2:**
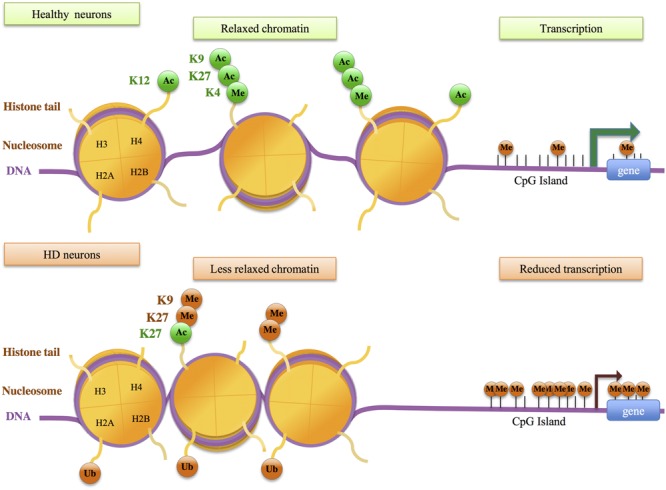
**Histone modifications and DNA methylation changes in Huntington’s disease (HD) neurons.** Genes in healthy neurons are enriched in transcriptionally active histone modifications, including acetylation of histone H3 at lysine 27 (H3K27ac), H3K9ac, H4K12ac, trimethylation of H3K4 (H3K4me3). Down-regulated genes in HD neurons show decreased levels of active histone modifications and increased levels of histone modifications associated with transcriptionnally repressed chromatin, including H3K9me2, H3K27me3, and ubiquitylation of H2A (H2A ub), resulting in less relaxed chromatin and decreased transcription. These events may be associated with increased DNA methylation at gene promoters.

Extensive changes in histone acetylation levels were observed in cellular systems based on mutant Htt overexpression (Steffan et al., 2001; Igarashi et al., 2003). However, global levels of H2B, H3 and H4 acetylation appeared unchanged between brain tissues of HD R6/2 and control mice (Hockly et al., 2003; Sadri-Vakili et al., 2007). Studies using the striatum of HD mice further suggested that decreased H3 acetylation occurs at selective gene loci, particularly at promoters of down-regulated genes such as *Drd2, Penk1, Actb*, or *Grin1* (Sadri-Vakili et al., 2007). Using chromatin immunoprecipitation paired with microarray hybridization (ChIP-chip), McFarland et al. (2012) assessed histone acetylation changes at genome-wide scale in the striatum of HD R6/2 transgenic mice. H3K9 and H3K14 acetylations (H3K9,14ac) and transcriptional changes between HD and WT striatum were poorly correlated in the striatum of HD R6/2 mice, suggesting that variation in H3K9,14ac levels alone may not be sufficient to account for gene expression changes in HD mice. Valor et al. (2013) reached similar conclusions by investigating changes in H3K9,14 ac and H4K12ac using a more powerful method –ChIPseq- on hippocampus and cerebellum of the HD transgenic N171-82Q mouse model (**Figure 2**). However, whereas data obtained by McFarland et al. (2012) suggest broad changes in histone acetylation in HD mouse striatum when compared to WT striatum, the results reported by Valor et al. (2013) indicate that changes are restricted to few loci. The absence of bulk changes in histone acetylation is further supported by a study showing promoter deacetylation of H3 at specific loci in HD models (Guiretti et al., 2016).

**FIGURE 3:**
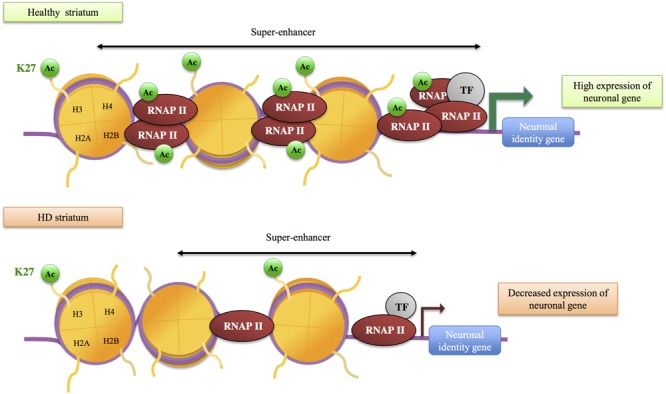
**Role of super-enhancers in gene down-regulation in HD.** Super-enhancers, a category of large enhancers enriched in H3K27ac, regulate transcription of neuronal identity genes in the striatum. In HD mouse striatum, H3K27ac and RNA polymerase II (RNAP II) are decreased at super-enhancers, which likely contributes to reduced transcription of neuronal identity genes. TF, transcription factor.

H3K27ac, a mark of active enhancers, was also selectively decreased in the striatum of HD R6/1 mice (Achour et al., 2015) (**Figure 2**). Integrating H3K27ac ChIPseq with RNA polymerase II (RNAPII) ChIPseq and RNAseq data, Achour et al. (2015) showed that H3K27ac and RNAPII are decreased at regions enriched in down-regulated genes, providing evidence for a strong correlation between decreased H3K27ac, decreased RNAPII and gene down-regulation in HD mouse striatum. Moreover, enhancer regions presenting reduced H3K27ac in HD R6/1 striatum were enriched in super-enhancers, a category of broad enhancers, regulating genes that define cell type-specific identity and function (**Figure 3**). In fact, striatal super-enhancer genes down-regulated in HD mouse striatum were enriched in genes controlling neuronal activity, including neuronal plasticity and transmission (Achour et al., 2015). Thus, the results suggest that selective decrease in super-enhancer activity underlies HD neuronal transcriptomic signature (e.g., down-regulation of genes that define neuronal identity and function, referred to as neuronal identity genes thereafter). This supports an epigenetic origin of gene down-regulation in HD.

##### Other histone modifications

The above results support the view that specific regulatory elements, super-enhancers, are sensitive to the HD mutation. Recent data indicate that selective promoters are also impaired in the striatum of HD mice and patients. Investigating H3K4me3, a mark of active promoters, down-regulated genes in the striatum and cortex of HD R6/2 mice were found to preferentially associate with broad promoters, regulating genes enriched in biological processes linked to neuronal function (Vashishtha et al., 2013). Moreover, H3K4me3 appeared decreased at down-regulated genes in R6/2 vs. WT tissues (**Figure 2**). Thus, these results show that specific broad promoter signature associates with decreased expression of neuronal genes in the striatum and cortex of HD mice. It is very likely that target genes of broad promoters and super-enhancers in brain tissues overlap and are enriched in neuronal identity genes. Moreover, in a study using ChIPseq on embryonic stem cells (ESC) and neural progenitor cells (NPC) expressing mutant Htt with various CAG sizes, a correlation was reported between CAG-repeat-dependent changes in gene expression and in H3K4me3 levels, particularly in NSC (Biagioli et al., 2015). This raises the hypothesis that mutant Htt might alter epigenetic regulation at early stage of neuronal differentiation. Finally, H3K4me3 has been investigated in the prefrontal cortex of HD patients using ChIPseq (Bai et al., 2015; Dong et al., 2015). In contrast to mouse data, human data indicate that epigenetic and transcriptional changes in HD *vs* control tissues are poorly correlated (Bai et al., 2015; Dong et al., 2015). However, in human experiments, sequencing depth appears suboptimal, which may have resulted in underestimation of H3K4me3 signals. Interestingly however, the study by Dong et al. (2015) indicates that promoters differentially enriched in H3K4me3 associate with genes involved in pathways or network linked to neuronal activity and inflammation, suggesting that transcriptional changes affecting inflammatory genes, in addition to those affecting neuronal genes, involve epigenetic mechanisms (**Figure 2**). The hypothesis that induction of inflammatory genes in HD neuronal tissues associates with loss of identity of glial cells would need to be investigated (Gabel et al., 2016).

Additional epigenetic modifications at regulatory regions might also be impaired. Increased levels of H3K9me2, a mark associated with heterochromatin, have been reported in the striatum of HD patients and R6/2 mice, using immunohistological analyses (Ryu et al., 2006). Whether specific loci are more particularly sensitive to increased H3K9 methylation in HD models has yet to be investigated using genome-wide approach. Moreover, the level of H3K27me3, a repressive histone modification that can mark promoters and enhancers, was modulated by CAG-repeat size in both ESC and NPC (Seong et al., 2010; Biagioli et al., 2015). However, the transcriptional consequences of this effect were unclear. Finally, H2A ubiquitylation (H2Aub) was found increased at down-regulated genes in HD R6/2 mice (Kim et al., 2008), and ChIP-on-chip analysis of H2Aub changes in R6/2 striatum indicated that histone changes were not restricted to dysregulated genes (McFarland et al., 2012).

##### DNA methylation

Genome-wide analysis of DNA methylation was performed in HD cell models (Ng et al., 2013). Changes in DNA methylation in response to mutant Htt were observed at both promoter proximal and distal regulatory regions. Interestingly, a large fraction of the genes that changed in expression upon mutant Htt expression displayed changes in DNA methylation, suggestive of a causal relationship (**Figure 2**). DNA methylation was also profiled using post-mortem cortex and liver from HD patients (De Souza et al., 2016). The results revealed minimal evidence of HD-associated DNA methylation. However, the HTT gene was methylated in a tissue-specific manner, which might lead to tissue-specific regulation of HTT promoter activity. Additionally, 5-hydroxymethylcytosine (5-hmC) and 7-methyl guanine (7-MG) were globally reduced in brain tissues of HD mouse models, including YAC128 mice (5-hmC study), R6/2 and CAG140 knockin mice (7-MG study) (Thomas et al., 2013; Wang et al., 2013). While these studies further provide evidence for altered DNA methylation in HD brain tissues, transcriptional consequences of such impairments remains elusive. Analysis of DNA methylation has also been used to assess the epigenetic clock of HD patients (Horvath et al., 2016). Horvath recently developed an epigenetic measure of tissue age, so-called epigenetic age, estimated from DNA methylation levels of 353 CpG sites (Horvath, 2013). The study using brain tissues from HD patients showed accelerated epigenetic aging in HD brain, particularly in cortical tissues. However, this was not the case for striatal tissues, possibly due to excessive neuronal loss (Horvath et al., 2016). While transcriptional significance of accelerated aging in HD brain is unclear, the data might reveal an age-dependent alteration of epigenetic regulation. Finally, treatment of HD mice with the HDAC inhibitor HDACi 4b led to transgenerational effects, possibly mediated by increased DNA methylation at CpG sites associated with Kdm5d (Jia et al., 2015). Thus, DNA methylation may be a therapeutic target for HD.

### Mechanisms Involved in Altered Epigenetic Regulation in HD

**FIGURE 4:**
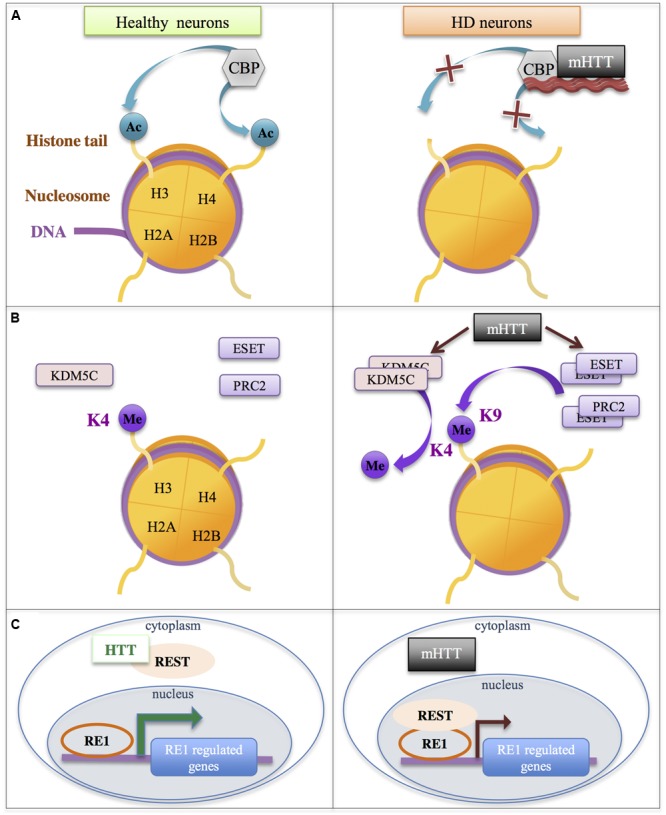
**Additional mechanisms involved in gene down-regulation in HD.** Mutant HTT (mHTT) leads to the modulation of the activity of chromatin modifiers/complexes (e.g., CBP, ESET, KDM5C, PRC2) and transcriptional regulators (e.g., REST). **(A)** The HAT CBP is sequestrated in mHTT aggregates and/or interacts with soluble mHTT, which reduces its activity, leading to reduction of histone acetylation. **(B)** Increased levels/activities of the H3K4me3 demethylase KDM5C, of the methyltransferase ESET and of polycomb repressive complex PRC2 lead to increased histone methylation. **(C)** HTT interacts with REST, limiting its availability in the nucleus. This interaction is impaired with mHTT, leading to increased binding of REST to RE1 elements and down-regulation of RE1-regulated genes. ESET, ERG-Associated Protein with SET Domain; HTT, Huntingtin; KDM5C, Lysine Demethylase 5C; PRC2, polycomb repressive complex 2; REST, RE1 Silencing Transcription Factor.

As mentioned above, altered histone acetylation in HD has been proposed to result from decreased activity of the HAT CBP, due to CBP recruitment into aggregates of mutant Htt, CBP depletion in neurons expressing mutant Htt and/or through an aberrant interaction of CBP with soluble mutant Htt (Steffan et al., 2000; Nucifora et al., 2001; Jiang et al., 2006; Seredenina and Luthi-Carter, 2012; Glajch and Sadri-Vakili, 2015) (**Figure 4**). The effect appears specific to CBP since CBP-related HAT p300 was unable to rescue cell toxicity in overexpression assays (Nucifora et al., 2001). However, although studies manipulating CBP levels in HD models support a role for CBP in pathogenesis (Steffan et al., 2000; Klevytska et al., 2010), including cognitive deficits (Giralt et al., 2012), it is still unclear whether altered CBP underlies HD neuronal transcriptional signature.

The activity of additional chromatin modulators was also found affected by Htt and/or mutant Htt. Htt was associated with polycomb repressive complex 2 (PRC2) in cell nucleus, which facilitated stimulation of PRC2 activity (**Figure 4**). Noticeably, CAG-expanded Htt further enhanced PRC2 activity in cell models (Seong et al., 2010). However, although H3K27me3 ChIPseq analyses revealed that Htt null mutation in ESC globally decreased H3K27me3, a result consistent with Htt-mediated stimulation of PRC2, the trend was opposite in NPC, suggesting that Htt may also be implicated in the process of H3K27me3 removal, and these different roles for Htt on H3K27me3 regulation may depend on cell differentiation state (Biagioli et al., 2015). Thus, the role of mutant Htt on genome-wide regulation of H3K27me3 needs to be further investigated, particularly in mature neurons. It is especially important to specify this issue since PRC2 deficiency in adult neurons leads to molecular, electrophysiological and behavioral phenotypes reminiscent to those seen in HD mice (von Schimmelmann et al., 2016). In fact, inactivation of PRC2 in striatal neurons resulted in re-expression of transcription factors involved in neuronal differentiation and in down-regulation of striatal identity genes, suggesting that transcriptional signatures resulting from PRC2 inactivation and from the expression of mutant Htt in the striatum share similarities (von Schimmelmann et al., 2016).

Additional enzymes modulating histone methylation were also deregulated in HD models. ESET/SETB1, a H3K9 methyltransferase, was increased in the striatum and cortex of HD R6/2 mice due to SP1/SP3-mediated transcriptional activation, which resulted in increased histone H3K9 methylation (Ryu et al., 2006) (**Figure 4**). Moreover, KDM5C/JARID1C, an enzyme involved in demethylation of H3K4me3, was up-regulated in the striatum and cortex of HD R6/2 mice and proposed to contribute to decreased H3K4me3 in HD brain tissues (Ng et al., 2013; Vashishtha et al., 2013) (**Figure 4**). Other studies further suggest that Htt and/or mutant Htt affect chromatin structure through modulation of the activity of enzymes regulating histone methylation, including H3K9 and H3K4 methylation (Ooi and Wood, 2007; Lee et al., 2013; Dietz et al., 2015).

As already mentioned, transcription factors also contribute to remodel the chromatin through the recruitment of histone modifying enzymes, including enzymes involved in histone acetylation and methylation. For instance, following binding to their cognate DNA response element, several transcription factors can interact with CBP in a stimulus-dependent manner, thereby increasing CBP concentration and histone acetylation at selective gene regulatory elements (e.g., enhancers and/or promoters) (Sterner and Berger, 2000). Thus, impaired level or activity of transcription factors in HD may also affect epigenetic regulations. Many transcription factors, including REST, SP1, TAF130, p53, were found deregulated in cells expressing the HD mutation, due to aberrant interaction with mutant Htt or altered transcriptional regulations (Nucifora et al., 2001; Bae et al., 2005; Ravache et al., 2010; Zuccato et al., 2010; Moumne et al., 2013; Langfelder et al., 2016) (**Figure 3**). It is tempting to speculate that some of these factors, particularly those regulating neuronal differentiation or maintenance of neuronal fate (this is for instance the case of REST), might contribute to HD epigenetic and transcriptional alterations.

Additionally, the ability of transcription factors to interact with chromatin-modifying enzymes depends in some cases on the activation of specific signaling pathways, which permits to optimize the coupling between epigenetic and transcriptional responses after stimulation. This is the case for CREB, which needs to be phosphorylated in response to activation of cAMP signaling pathway, to bind and recruit CBP on chromatin (Cardinaux et al., 2000). The Ras/MAPK/MSK1 signaling pathway is another pathway, where transcriptional and epigenetic responses are coordinated through phosphorylation events targeting transcription factors and histones (e.g., ELK1 and H3 at serine 10, respectively) (Brami-Cherrier et al., 2009; Bahrami and Drablos, 2016). These pathways, which control the dynamics of IEGs expression such as *Fos, Egr1*, and *Arc*, play a key role in the regulation of neuronal activity, including plasticity and excitability (Besnard et al., 2011; West and Greenberg, 2011; Lopez-Atalaya and Barco, 2014). Several studies suggest that these pathways are impaired in HD striatum, raising the hypothesis that the coupling between epigenetic and transcriptional mechanisms regulating neuronal activity is altered in HD (Besnard et al., 2011; Langfelder et al., 2016), which might contribute to altered epigenetic regulations (Chawla et al., 2003).

Despite these hypotheses, it remains unclear how mutant Htt leads to selective alteration of the chromatin associated with neuronal identity genes. The underlying mechanism may be the addition of direct effects of mutant Htt on the chromatin and transcription regulators described above (e.g., CBP, PRC2, ESET/SETB1, KDM5C/JARID1C, REST, SP1, CREB, TAF130, p53). Alternatively, it may be the result of an indirect mechanism caused by mutant Htt on neuronal homeostasis and activity that is yet to be identified.

## Emerging Picture and Perspectives

Here, we reviewed the current understanding of the role of neuroepigenetics in HD, in an attempt to uncover the significance of epigenetic changes in HD brain, a prerequisite to the rational design of epigenetic therapies.

Three main questions underlie the review: (1) What are epigenetic changes in HD? (2) What is the consequence of epigenetic changes in HD, particularly on transcriptional regulation? (3) What mechanism(s) cause(s) epigenetic changes in HD? Although, we still lack complete answers to any of these questions, an emerging picture arises, supporting the view that maintenance and/or establishment of neuron-specific chromatin identity is altered in HD, which leads to down-regulation of neuronal identity genes. This suggests that mutant Htt might interfere with neuronal differentiation, in agreement with recent studies (Molero et al., 2016). To investigate this hypothesis, it will be crucial to assess HD epigenome and transcriptome across time, including developmental stages. If altered epigenetic regulation in HD brain results in down-regulation of neuronal identity genes, it is very likely to disturb neuronal activity, including synaptic plasticity and neuronal excitability, controlling learning and memory processes. This will need to be investigated. The issue of tissue-/cell-specificity of epigenetic alterations in HD also remains to be investigated. It is unclear whether the HD mutation induces similar epigenetic changes between cell types (e.g., neurons vs. glial cells) or between different tissues (e.g., striatum vs. cortex, hippocampus or cerebellum, neuronal tissue vs. non-neuronal tissue). It will also be crucial to investigate the timing of establishment of super-enhancer signatures relative to transcriptional changes to specify the relationship between epigenetic and transcriptional changes in HD brain tissues. Addressing these questions will certainly provide insights into the mechanism causing epigenetic and transcriptional changes in HD, which may be a key to the identification of new therapeutic targets.

Comprehensive analysis of the HD epigenome using various HD models and genome-wide techniques, including techniques that permit to investigate epigenomes at cell type-specific or single-cell scale and in 3D (using chromosome conformation capture- (3C)-based methods) is necessary to address these questions. Another challenge will be to develop bioinformatics methods to analyze the data and extract meaning. This may require the development of approaches that reduce complexity, such as network-based methods (Langfelder and Horvath, 2008; Parmentier et al., 2013).

Epigenetic therapies to treat neurodegenerative diseases have been first considered for HD, when Steffan et al. (2000) showed that HDAC inhibitors improve the phenotype of HD flies. In fact, preclinical studies to evaluate the effect of HDAC inhibitors in HD have inspired other neurodegenerative diseases, including additional trinucleotide repeat (TNR) diseases (for review see Evans-Galea et al., 2013) PD, Alzheimer disease (AD), and amyotrophic lateral sclerosis (ALS) (Xu et al., 2011; Lazo-Gomez et al., 2013; Sharma and Taliyan, 2015a). Beneficial effects have been observed in preclinical models for these different diseases, though the effects of HDAC inhibitors appear partial and underlying mechanisms are unclear (Benito et al., 2015; Harrison et al., 2015). In the specific case of TNR diseases, HDAC inhibitors may affect disease progression through two independent mechanisms: (1) through a general effect on gene expression program, (2) through modulation of the stability of TNRs, which are subject to epigenetic regulations (Goula et al., 2012; Evans-Galea et al., 2013). To overcome limitations associated with the use of HDCAi, including the lack of specificity and toxicity, HDACi-alternative epigenetic therapies are currently being developed that are based on HAT activators, histone methyltransferase inhibitors and DNMT modulators (**Table 1**).

**Table 1:** Examples of histone deacetylase (HDACi)-alternative epigenetic therapies.

Drug family	Target	Molecules	Mechanisms and effects	Reference
HAT activators	p300/CBP	N-(4-chloro-3-trifluoromethyl-phenyl)-2-ethoxy-6-pentadecyl-ben-zamide (CTPB)	CTPB leads to the neurite growth of a PD cell model, and protects them from cell death induced by the neurotoxin 6-hydroxydopamine.	Hegarty et al., 2016
	p300/CBP	CSP-TTK21	CSP-TTK21 promotes differentiation and maturation of young adult hippocampal neurons and improves long-term retention of a spatial memory.	Chatterjee et al., 2013
HAT inhibitors	p300	C646	C646 reduces amount of Tau and neurotoxicity in culture rat neurons. C646 enhances fear extinction memory and synaptic plasticity.	Min et al., 2010
Histone methyltransferase inhibitors	EZH2	3- deazaneplanocin A (DZNep)	DZNep reactivates silenced genes in cancer cells and developmental genes that are not silenced by DNA methylation. DZNep inhibits H3K27me3 and H4K20me3.	Miranda et al., 2009
DNA methyltransferase inhibitors	DNMT1&3	Nucleoside analog: 5-fluoro-2’-deoxycytidine (FdCyd)	FdCyd has neuroprotective effects against mutant Htt-induced toxicity in primary cortical neurons in cell viability and neurite degeneration assays.	Pan et al., 2016
	DNMT1&3	Non-nucleoside inhibitor : RG108	RG108 is a DNMT active site inhibitor. RG108 blocks the increase in 5-methycytosine and prevents cell death in a mouse model of motor neuron disease model.	Chestnut et al., 2011
DNA methyltransferase activators	DNMT	PARP	Loss of nucleolar PARP-1 results in DNA methyltransferase activation. This may impact on ribosomal DNA silencing observed in AD.	Zeng et al., 2016
	DNMT1	Epstein-Barr virus latent membrane protein 1 (LMP1)	LMP1 directly induces the dnmt1 promoter activity through its COOH-terminal activation region-2 YYD domain.	Tsai et al., 2006; Li J. et al., 2016

*CBP, CREB-binding protein; EZH2, Enhancer of Zeste Homolog 2; DNMT, DNA MethylTransferase; HAT, Histone AcetylTransferase.*

Epigenomic studies on HD models might benefit from studies in other neurodegenerative diseases and reciprocally. Indeed, the similarity of epigenomic and transcriptomic signatures between HD and AD models (e.g., neuronal and inflammation signatures) is intriguing and might suggest that common epigenetic mechanisms to several neurodegenerative diseases might operate (Gjoneska et al., 2015).

## Author Contributions

LF and KM developed the literature review. KM, LF, and EB wrote the manuscript. LF and CL built the figures. All the authors read and commented the article throughout the writing, and collaborated in the revision of the final manuscript before submission.

## Conflict of Interest Statement

The authors declare that the research was conducted in the absence of any commercial or financial relationships that could be construed as a potential conflict of interest.
